# Application of High-Z Nanoparticles to Enhance Current Radiotherapy Treatment

**DOI:** 10.3390/molecules29112438

**Published:** 2024-05-22

**Authors:** Nolan Jackson, Daniel Cecchi, Wayne Beckham, Devika B. Chithrani

**Affiliations:** 1 Department of Physics and Astronomy, University of Victoria, Victoria, BC V8P 5C2, Canada; 2 British Columbia Cancer-Victoria, Victoria, BC V8R 6V5, Canada; 3 Centre for Advanced Materials and Related Technologies, Department of Chemistry, University of Victoria, Victoria, BC V8P 5C2, Canada; 4 Division of Medical Sciences, University of Victoria, Victoria, BC V8P 5C2, Canada; 5 Department of Computer Science, Mathematics, Physics and Statistics, Okanagan Campus, University of British Columbia, Kelowna, BC V1V 1V7, Canada

**Keywords:** nanoparticle, radiotherapy, radiosensitizer, cancer, chemotherapy, therapeutics

## Abstract

Radiotherapy is an essential component of the treatment regimens for many cancer patients. Despite recent technological advancements to improve dose delivery techniques, the dose escalation required to enhance tumor control is limited due to the inevitable toxicity to the surrounding healthy tissue. Therefore, the local enhancement of dosing in tumor sites can provide the necessary means to improve the treatment modality. In recent years, the emergence of nanotechnology has facilitated a unique opportunity to increase the efficacy of radiotherapy treatment. The application of high-atomic-number (Z) nanoparticles (NPs) can augment the effects of radiotherapy by increasing the sensitivity of cells to radiation. High-Z NPs can inherently act as radiosensitizers as well as serve as targeted delivery vehicles for radiosensitizing agents. In this work, the therapeutic benefits of high-Z NPs as radiosensitizers, such as their tumor-targeting capabilities and their mechanisms of sensitization, are discussed. Preclinical data supporting their application in radiotherapy treatment as well as the status of their clinical translation will be presented.

## 1. Introduction

Although massive strides in cancer therapeutics have been made over the years, cancer remains one of the leading causes of death worldwide. This has prompted continuous research to advance therapeutic options for this life-threatening disease, causing the landscape of cancer treatment to constantly evolve to improve patient outcomes. As a result, modern therapeutic cancer treatments, such as immunotherapy, targeted therapy, photodynamic therapy, etc., have emerged recently. However, the three main cornerstone treatment options, radiotherapy, surgery, and chemotherapy, still maintain their indispensable role in the management of patients’ diseases, continually benefitting from new medical and technological advancements to enhance the efficacy of each of the treatment options.

Currently, radiotherapy is a pivotal treatment modality used in the management of cancer. It is suggested that approximately 50% of cancer patients benefit from receiving radiotherapy, either as an independent treatment option or used in conjunction with surgery and/or chemotherapy, making it an essential component of treatment regimens for many patients [1]. In radiotherapy, ionizing radiation, such as high-energy X-rays, electrons, protons, neutrons, or light ions, transverse through a patient to locally target and damage cancer cells, with each type of ionizing radiation varying in its biological effects [2]. This offers a strategic advantage in treating deep-seated tumors that would otherwise be challenging to treat via conventional surgical methods.

While radiotherapy has proven to be invaluable due to its precision in targeting and killing tumor cells, it also presents some challenges. A major limitation in the overall efficacy of the treatment stems from the inevitable normal tissue toxicity that is induced. Despite radiotherapy being a highly localized, the ionizing radiation transverses through the patient to reach the target, so a portion of the dose is deposited in healthy tissue. This can lead to adverse effects and prevents the dose escalation that is required to enhance tumor control. Addressing this delicate balance between tumor control and the sparing of healthy tissue is an important problem in radiotherapy, which has led to advanced treatment delivery techniques, such as intensity-modulated radiotherapy (IMRT) and volumetric-modulated arc therapy (VMAT), that enable the delivery of highly conformal dose distributions, minimizing the dose delivered to normal tissues [3,4]. However, another therapeutic avenue that is currently being pursued to increase the efficacy of radiotherapy is the incorporation of radiosensitizers to radiotherapy treatment. By supplementing tumor sites with radiosensitizers, the radiation response of tumors can be enhanced, increasing the efficacy of the modality.

In general, radiosensitizers are agents that augment the sensitivity of cells to radiation. Their integration to increase the susceptibility of tumor cells to radiation-induced damage can help to mitigate challenges inherent to radiotherapy treatment, such as normal tissue toxicity and tumor radiation resistance [5]. For example, the tumor microenvironment can become hypoxic, increasing the resistance of tumors to radiotherapy treatment [6]. As a result, specific molecular agents have been developed that can elevate the oxygen levels within the tumor microenvironment, offsetting hypoxia and thereby increasing the sensitivity of tumors to radiation [7]. Furthermore, other radiosensitizing agents aim to target and inhibit certain cellular mechanisms that render cancer cells more prone to radiation damage, such as DNA repair pathways [8]. One of the primary concerns with radiosensitizers is the lack of specificity of some agents, which can inadvertently increase the toxicity in healthy tissues, creating a demand for novel radiosensitizers that can offer greater specificity, and in recent years, nanoparticles have emerged as a promising solution.

**Figure 1:**
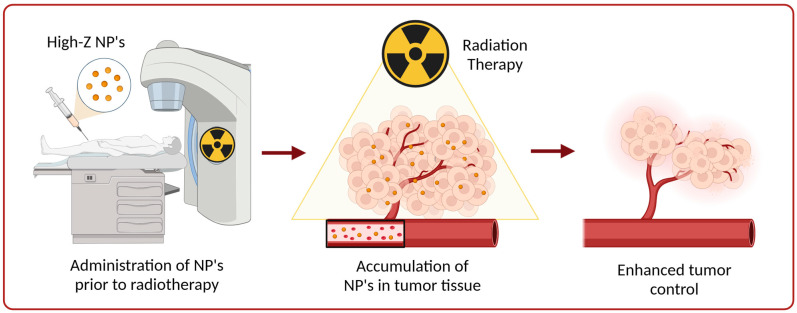
Idea for using radiosensitizing high-Z NPs in radiotherapy treatment regimens. Prior to radiotherapy, high-Z NPs can be administered to the patient and allowed to accumulate in tumor tissue. This can increase the tumor’s sensitivity to radiotherapy, leading to enhanced tumor control for patients. (Created using Biorender.com).

Due to the rapid advancements and developments in nanotechnology, the application of nanoparticles in cancer therapeutics has garnered much interest, offering innovative approaches to improve different aspects of cancer treatment. Nanoparticles (NPs), characterized by their size (typically ranging from 1 to 100 nanometres), possess unique physical and chemical properties at the nanoscale that allow them to be engineered and tailored for specific applications [9]. One of these applications is their use as radiosensitizers. Various high-atomic-number (Z) NPs have shown promising radiosensitizing effects and can be functionalized in ways such that they preferentially target tumor cells in comparison with normal tissue [10]. Through various mechanisms of radiosensitization, introducing high-Z NPs into the tumor environment can locally enhance the therapeutic effects of radiotherapy (Figure 1) without introducing additional normal toxicity. Furthermore, high-Z NPs can serve as a targeted drug delivery system for drug-based radiosensitizing agents, serving as a bi-functional radiosensitizing NP. Currently, the spectrum of high-Z NPs that are being investigated for this purpose are, but not limited to, gold (Z = 79), silver (Z = 47), bismuth (Z = 83), gadolinium (Z = 64), and hafnium (Z = 72).

This article will encompass the therapeutic benefits of implementing high-Z NPs into radiotherapy, such as their targeting capabilities and mechanisms of sensitization. The preclinical clinical data supporting their use and the current clinical status of their application in radiotherapy treatment will also be discussed.

## 2. Tumor-Targeting Capabilities of Nanoparticles

The tumor-targeting capabilities of NPs are instrumental in their role as promising radiosensitizers. When exploring new strategies to improve radiotherapy, enhancing dose delivery to tumor sites is critical to increase the treatment’s therapeutic index. NPs possess this ability through their proficient tumor targeting capabilities, thereby increasing the local dose deposition in tumors relative to normal tissue.

The ability to modify the size and surface characteristics of NPs has enabled the ability to tailor them to preferentially accumulate within tumor tissue over normal tissues, leveraging both passive and active targeting mechanisms. In passive targeting, due to their small size, NPs can preferentially accumulate in tumor tissue via the enhanced permeability and retention (EPR) effect [11]. Conversely, active targeting uses the precision of molecular recognition, where the strategic functionalization of NPs with specific targeting for ligands or molecules allows them to selectively bind to cancer cell-specific markers [12]. By utilizing these two targeting strategies, NPs can be optimized to increase their therapeutic impact as radiosensitizers.

### 2.1. Passive Targeting

**Figure 2:**
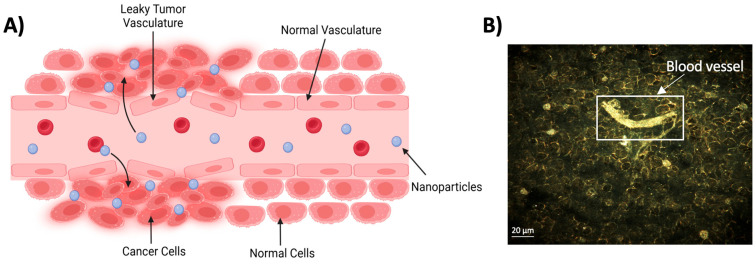
Passive targeting of NPs. (**A**) The poor structural integrity and lymphatic drainage of the tumor vasculature system result in the passive preferential accumulation of NPs in tumors compared with normal tissue. This is referred to as the EPR effect. (Created using Biorender.com). (**B**) Hyperspectral imaging demonstrating blood vessels facilitating the delivery of GNPs to the surrounding mice tumor tissue. Reproduced with permission from open access Creative Common license [16].

The premise of the passive targeting of NPs within tumor tissue is due to the EPR effect. The EPR effect is a phenomenon in which small molecules and particles tend to preferentially accumulate within tumor sites compared with normal tissue. This selective accumulation stems from the chaotic nature of the vasculature system present within the tumor microenvironment that forms as a result of angiogenesis, a hallmark of cancer. As tumors begin to rapidly proliferate, it necessitates the formation of new blood vessels to support the growth of the tumor. However, the neovasculature system often has structural abnormalities, consisting of improper endothelial junctions that permit the seepage of small molecules into tumors [13]. Additionally, the poor lymphatic drainage and slow venous return characterized in tumors impede the removal of these small molecules, promoting their retention [14,15]. This facilitates an increase in the accumulation of small molecules in tumor tissue compared with normal tissue, in which the normalized vasculature system does not exhibit these same characteristics (Figure 2). Therefore, the diminutive nature of NPs makes them ideal candidates to exploit this phenomenon to enhance their delivery to tumor sites.

In order to take full advantage of the EPR effect, prolonging the blood circulation time of NPs to allow them adequate time to reach tumors is a critical aspect that needs to be considered. This is commonly achieved through the addition of ‘stealth’ molecules, like polyethylene glycol (PEG), to the surface of NPs, which enables them to evade clearance from the reticuloendothelial system, preventing opsonization and subsequent phagocytosis [17,18]. As NPs enter the bloodstream, opsonin molecules can adsorb onto the surface of the NP, effectively tagging it for clearance via phagocytosis. The PEGylation of NPs offers a protective hydrating layer that minimizes protein corona adsorption, thus reducing their recognition by phagocytes and effectively extending their presence within the bloodstream. Unfortunately, this has also been shown to alter the NP uptake efficiency of cancer cells. Studies have reported that the addition of PEG to gold NPs (AuNPs/GNPs) resulted in a significant decrease in cellular uptake, which is attributed to the PEG moiety interfering with NPs from successfully interacting with and binding to tumor cell receptors [19]. To remediate this, further surface modification of NPs is necessary to promote tumor targeting, which can be achieved through active targeting mechanisms.

### 2.2. Active Targeting

**Figure 3:**
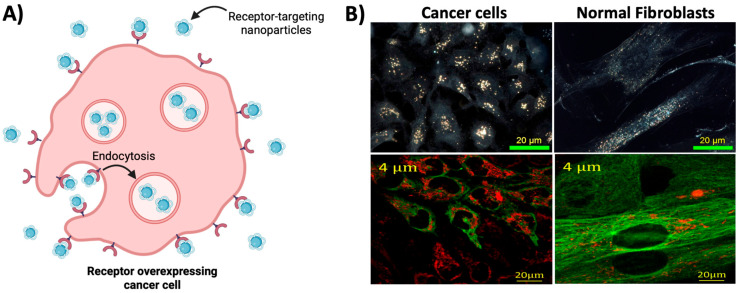
Active targeting of NPs. (**A**) Many cancer cells overexpress various surface receptors in comparison with normal cells. The surface of NPs can be strategically modified with corresponding ligands to specifically bind to these receptors, increasing their uptake in cancer cells. (Created using Biorender.com). (**B**) Accumulation of cancer targeting GNPs in cancer cells versus normal fibroblasts. Cancer cells demonstrate greater accumulation of GNPs compared with normal fibroblasts due to their overexpression of targeted receptors. Top panel: hyperspectral imaging of GNPs. Bottom panel: confocal imaging of GNPs (red) and microtubules (green). Reproduced with permission from open access Creative Common license [20].

The active targeting capability of NPs is a key feature that has propelled the application of NPs as radiosensitizers. Unlike passive targeting, which relies on the pathophysiological properties of tumor tissue, active targeting aims to strategically functionalize NPs with specific targeting moieties that are selectively recognized by tumor cells. This strategy aims to exploit the overexpression of certain surface receptors that are exhibited by cancer cells, where the overexpressed surface receptors can be targeted by NPs conjugated with corresponding ligands that results in the preferential attachment and subsequent cellular internalization via receptor-mediated endocytosis (Figure 3). Examples of such targeting ligands that are being investigated include peptides, aptamers, antibodies, and sugar molecules. The following section will provide examples of targeting strategies that are currently being used.

#### 2.2.1. Antibody-Coated NPs

Human epidermal growth factor receptor 2 (HER2) is a transmembrane tyrosine kinase receptor that regulates cell growth and differentiation, with many breast cancer patients (20–30%) presenting an amplified expression of the protein [21]. It has also been identified to be a suitable marker for poor prognosis, where the inhibition of HER2 has been shown to increase the sensitivity of radioresistant breast cancer cells to radiotherapy [22]. HER2 has been extensively studied as a therapeutic target, and an FDA-approved monoclonal antibody, Trastuzumab, has been shown to be effective in treating patients with HER2-overexpressing breast cancer [23]. Trastuzumab selectively binds to HER2 receptors, preventing them from receiving growth signals and thereby reducing cell proliferation. This selectivity of Trastuzumab has led researchers to conjugate the antibody to NPs to enhance their targeting effects towards cancer cells overexpressing HER2. Using trastuzumab as a targeting antibody, Cai et al. engineered a AuNP platform to enhance the targeted delivery of ^111^In, a radionuclide emitting low-energy (<25 keV) Auger electrons, in addition to its γ-emissions (Eγ = 171 keV (90%) and 245 keV (94%)) [24]. Their results showed that Trastuzumab-functionalized AuNPs were more efficiently internalized by HER2-positive cell lines, SK-BR-3 and MDA-MB-361, compared with their unfunctionalized counterparts. Furthermore, this targeted therapeutic strategy was proven to be effective in vivo, where the absorbed dose deposited in tumor tissue was 60.5 Gy compared with normal organs that received <0.9 Gy. More recently, affibodies that target HER2 have been developed and tested as targeting molecules. Affibody molecules are small proteins that can selectively bind to target proteins, essentially mimicking monoclonal antibodies. However, affibodies are much smaller, have a lower cost of production compared with monoclonal antibodies, and have high affinity and high tolerance to chemicals, higher temperatures, and extreme pH values [25]. A study conducted by Pourshohod et al. demonstrated their effectiveness, where AuNPs functionalized with an affibody that recognizes HER2 (Z_HER2_) increased the sensitivity of HER2-positive cell lines to radiation compared with non-functionalized AuNPs [26]. They further corroborated these findings in another study that demonstrated enhanced sensitization due to silver NPs (AgNPs) functionalized with the Z_HER2_ affibody [27].

**Figure 4:**
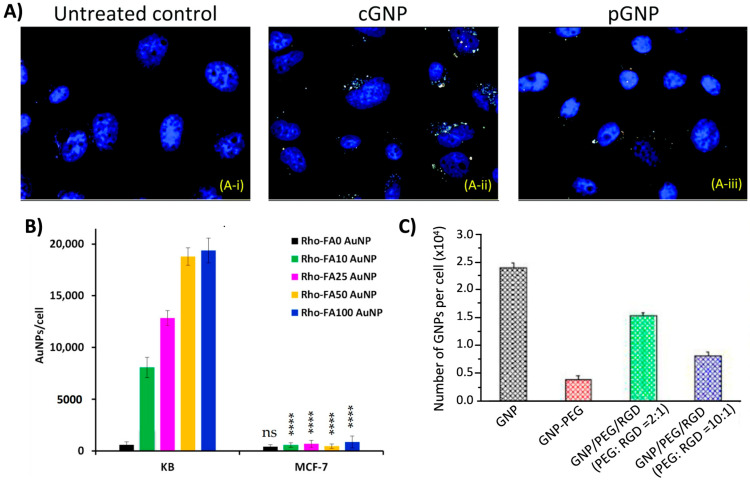
Enhanced cellular uptake of nanoparticles via targeting moieties. (**A**) Dark-field imaging of gold nanoparticles (yellow color) in MDA-MB-231 cells incubated with PBS (control), anti-CXCR4 functionalized GNPs (cGNP), or PEGylated GNPs (pGNP). Cell nuclei are stained in blue. (**B**) Cell uptake of rhodium-labeled folate functionalized AuNPs (Rho-FAx-AuNPs) by KB (overexpression of folate receptors) and MCF-7 (low expression of folate receptors) cells. AuNPs were functionalized with various surface concentrations of folate. Statistical significance was calculated versus non-targeted particles (Rho-Fa0 -AuNPs): **** *p* < 0.0001, ns nonsignificant. (**C**) Effects of PEG and RGD on the cellular accumulation of GNPs. Reproduced with permission from open access Creative Common license [16,28,30].

Similar to HER2, CXC chemokine receptor 4 (CXCR4) is commonly overexpressed by most breast cancers, making it an attractive targeting option for the treatment of breast cancers. One of the benefits of targeting CXCR4 is that this receptor is still expressed by triple-negative breast cancer, characterized by the absence of estrogen receptors (ER), progesterone receptors (PR), and HER2. Bhattarai et al. highlighted the prospect of targeting CXCR4 to enhance the efficacy of GNPs as radiosensitizers [28]. In their study, PEGylated GNPs were targeted towards this receptor via conjugation to an anti-CXR4 antibody. Dark-field images confirmed that the functionalized anti-CXCR4 GNPs (cGNP) resulted in enhanced uptake by cells compared with the PEGylated GNPs (pGNP) (Figure 4A). Furthermore, when combined with radiotherapy, it was found that the efficacy of the treatment strategy was dependent on the CXCR4 expression of various cell lines, displaying the dependence of receptor expression for targeting and treatment efficacy.

#### 2.2.2. Folate-Coated NPs

Folic acid or folate is an essential molecule for various crucial cellular processes, such as DNA synthesis and repair, RNA synthesis, amino acid metabolism, and phospholipid biosynthesis, making it an effective targeting ligand for the application of targeted cancer therapeutics. Due to the rapid division and proliferation of cancer cells, they often have an increased demand for compounds required for DNA synthesis. This results in the upregulation of folate receptors on the surface of cancer cells in attempts to receive an increased supply of folate from the surrounding environment to meet the high demand necessary to support the heightened growth rate of tumors. Therefore, folic acid is a prevalent targeting molecule to enhance tumor selectivity, and it has been used for NP targeted applications, facilitated by its favorable conjugation chemistry to the surface of NPs [29]. A study conducted by Daniele et al. investigated the targeting efficiency of folic acid, comparing the cellular internalization of PEGylated AuNPs functionalized with folic acid (FA AuNPs) versus non-functionalized PEGylated AuNPs [30]. Their results displayed that the accumulation of FA AuNPs was significantly increased in folate receptor-overexpressing KB cells compared with their unfunctionalized counterparts, with the increase being dependent on the AuNP surface density of folate (Figure 4B). However, the internalization of AuNPs was significantly less in MCF-7 cells that exhibit low expression of folate receptors, highlighting the selective targeting of folate-functionalized NPs. These targeting capabilities of folic acid were further demonstrated by Kefayat et al. using a C6 glioma tumor model [31]. Folic acid and BSA-decorated gold nanoclusters (FA-AuNCs) were shown to have 2.5 times higher cellular accumulation in C6 cancer cells compared with L929 normal cells, effectively displaying the preferential selectivity towards cancer cells. This was shown to translate in vivo with a higher concentration of FA-AuNCs localized in brain tumors compared with the surrounding normal tissue. When combined with a radiation dose of 6 Gy, the median survival of tumor-bearing mice increased from 18 days (radiotherapy alone) to 24.5 days (radiotherapy + FA-AuNPs), indicating the applicability of folic acid-coated NPs as promising radiosensitizers.

#### 2.2.3. Peptide-Capped NPs

Commonly used targeting ligands are peptides containing the arginine–glycine–aspartic acid (RGD) sequence. RGD exhibits a high affinity for the integrin receptor α_ν_β_3_, an integrin receptor that is highly overexpressed by many solid tumors. The ability of RGD to effectively target various forms of cancer has made it a versatile tool in oncological applications, which has extended to its application in NP-mediated therapeutics. Highlighting this, research by Cruje et al. demonstrated that PEGylated GNPs functionalized with RGD displayed enhanced GNP uptake by MDA-MB-231 breast cancer cells compared with unfunctionalized PEGylated GNPs [32]. Interestingly, this effect was only realized for GNPs with a diameter of 14 nm, but not for larger GNPs with a diameter of 74 nm. This was attributed to the smaller NPs having an increased surface curvature that aided the RGD peptide in accessing the integrin receptors on the surface of the cells, suggesting that parameters such as NP size must be taken into consideration when functionalizing NPs for targeting purposes. Additionally, Yang et al. found similar results in MIA-PaCa-2 cells, with RGD significantly enhancing the cellular uptake of GNPs, where an increase in RGD density resulted in a greater accumulation of GNPs (Figure 4C) [16]. This correlation between RGD density and cell accumulation also extended to in vivo environments, with a greater density yielding enhanced tumor accumulation and retention, demonstrating the tumor-targeting abilities of RGD-functionalized NPs.

## 3. Mechanisms of Radiosensitization

### 3.1. Physical Mechanism of NP-Based Radiosensitization

The deposition of a dose of ionizing radiation is facilitated through the transfer of its energy to absorbing materials, and in the case of ionizing photons, this transfer of energy primarily occurs via ionizing interactions. The primary ionizing interactions through which incident photons in the keV to low MeV range lose energy are the Compton and photoelectric effects. In the Compton effect, incident photons are scattered due to collisions with weakly bound electrons, transferring a portion of their energy that results in the electron being ejected from the atom. These photons, which retain most of their energy, have long ranges, leading to a sparse distribution of ionizing events. In the case of photoelectric interactions, electrons from the inner atomic orbitals absorb the incident photons, causing the ejection of these inner-shell electrons. The resultant inner shell vacancy is subsequently filled by an outer shell electron, thereby releasing energy that causes the emission of low-energy fluorescent photons and a cascade of secondary electrons known as Auger electrons. These Auger electrons are low energy, depositing their energy in close proximity to the absorbing material with an ionizing track ranging up to a few micrometers, resulting in highly localized ionizing events and enhancing local dose deposition [33].

**Figure 5:**
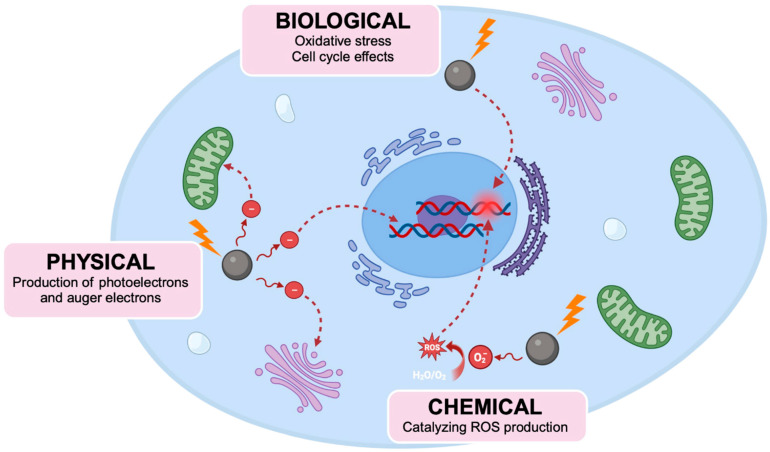
Mechanisms of high-Z NP radiosensitization. NPs can increase the sensitivity to radiation of cells through physical, chemical, and biological mechanisms. NPs interact with ionizing radiation, producing a shower of secondary electrons that can exert damage upon the cell. The electronically active surface NPs also catalyze the production of DNA-damaging ROS and free radicals, damaging critical cellular components. NPs can also increase the sensitivity of cells to radiation through oxidative stress and cell cycle effects. (Created using Biorender.com).

The physical mechanism of NP-mediated radiosensitization primarily hinges on the difference in energy absorption properties between soft tissue (Z~7.3) and high-Z NPs, arising from the difference in atomic number between the two absorbing materials. While the X-ray cross-section (probability of interaction) for the Compton effect is largely independent of the atomic number of the absorbing material, the X-ray cross-section for the photoelectric effect is approximately proportional to Z^3^–Z^4^. Consequently, this leads to an increase in ionizing interactions, particularly the photoelectric effect, for high-Z-absorbing materials. By incorporating high-Z NPs, such as gold (Z = 79), silver (Z = 47), bismuth (Z = 83), and gadolinium (Z = 64) into tumor sites, they can provide a significantly greater amount of energy per unit mass due to their high energy absorption compared with that of soft tissue, thus enhancing the local deposition of dose in tumors (Figure 5).

However, the extent to which this physical dose enhancement contributes to the observed radiosensitization of cells remains unclear. The increased photoelectric interactions by high-Z NPs are more pronounced for incident photons produced by kilovoltage (kV) X-ray sources, where the strong photoelectric absorption at lower keV energies leads to a dramatic increase in the absorbed dose [34,35]. In contrast, for photons produced by megavoltage (MV) X-ray sources, which is prevalent in most clinical scenarios, the primary interaction is the Compton effect, where the X-ray cross-section is essentially independent of atomic number. Thus, it was thought that the differential in dose deposition due to the incorporation of high-Z NPs would be insignificant when using MV X-ray sources [36,37]. Interestingly, in vitro and in vivo experimental evidence indicates radiosensitization effects that often surpass the expected physical dose enhancement. For instance, a study conducted by Chithrani et al. demonstrated that HeLa cells exposed to GNPs with a diameter of 50 nm exhibited a radiosensitization-enhancement factor (REF) varying with the energy of the X-ray sources (1.66 with 105 kVp, 1.43 with 220 kVp, and 1.17 with 6 MV) [38]. Although their study effectively displayed the energy dependence of the radiosensitization due to GNPs, it suggested that GNPs were effective in increasing the radiosensitization of cells when treated with MV X-rays. These results have also been further corroborated by various studies that effectively display the sensitizing effects of high-Z NPs when irradiated by MV sources, indicating that other mechanisms could be responsible for the observed radiosensitization [20,39,40,41,42].

### 3.2. Production of Reactive Oxygen Species (ROS) and Oxidative Stress

One of the potential mechanisms of NP-based radiosensitization is attributed to the production of reactive oxygen species (ROS) (Figure 5). The production of ROS and the subsequent oxidative stress is an important pathway that is responsible for cellular damage during radiotherapy. When exposed to radiation, water molecules within the cell undergo radiolysis that subsequently leads to the production of ROS, such as superoxide anion radicals (O_2_^−^), hydroxyl radicals (OH^−^), and hydrogen peroxide (H_2_O_2_). These reactive species can directly damage DNA, triggering various cellular responses, including cell death. In addition, the excessive production of ROS can exert oxidative stress within the cell as a result of ROS levels surpassing the reducing capacity of the cell. This can impair various cellular functions due to the oxidation of proteins and lipids, and damage induced to the mitochondria, which can ultimately lead to apoptosis.

Numerous studies have shown that NPs can enhance ROS production following radiotherapy treatment. A study conducted by Misawa et al. demonstrated this concept, where the addition of AuNPs significantly enhanced the production of ROS in a water environment, with their results displaying 1.46- and 7.68-fold increases in the production of OH^−^ and O_2_^−^ radicals, respectively, under 100 kVp irradiation [43]. They attributed the rise in O_2_^−^ radicals to the electron transfer from the photo- and auger electrons, whereas the increased production of OH^−^ radicals was attributed to the secondary radiolysis of water by fluorescent X-rays, suggesting that high-Z NPs facilitate a significant production in ROS under irradiation. Furthermore, this enhancement in ROS has been demonstrated to translate in cell cultures for various NPs [44,45,46,47,48]. For instance, Bemidinezhad et al. found that glucose-coated AuNPs significantly increased the ROS levels in cell cultures post-irradiation, resulting in an upregulation in caspase 3/7 activity, crucial enzymes in the apoptosis pathway, which was correlated with an increase in the therapeutic response to radiotherapy treatment [49].

Interestingly, NPs have also been shown to amplify ROS production in the absence of radiation [50,51,52,53]. It is thought that NPs with catalytic surfaces can catalyze chemical reactions and increase the production of ROS, where radiation further enhances this effect due to the highly reactive environment. For example, while it was once widely accepted that AuNPs are chemically inert, evidence suggests that the surface of AuNPs is electronically active and can facilitate the surface-mediated transfer of electrons from electron donors, interacting with molecular oxygen to generate ROS such as superoxide radicals [54]. However, contrary evidence from a recent study has suggested that, although AuNPs catalyze the reduction of O_2_, the production of ROS is merely transient surface products produced as intermediate products in the reduction process, not contributing to ROS reactivity [55]. Rather, the oxidative stress observed in cells could be due to AuNPs catalyzing redox reactions, subsequently oxidizing antioxidants and reducing the redox capacity of cells, eventually leading to oxidative stress.

This reduction in the redox capacity of cells mediated by the introduction of NPs to the cellular environment is a mechanism that has been previously explored. One of the key mechanisms underlying this effect is through the inhibition of endogenous reducing agents, such as glutathione (GSH), an essential molecule in maintaining the redox homeostasis of cells. NPs can interact with reducing agents, leading to their depletion or inactivation. For example, NPs that have a high affinity for thiol groups can bind to antioxidants containing thiol groups, such as GSH, effectively inhibiting the reducing ability of the molecule and thereby decreasing the reduction capacity of the cell [56]. A study conducted by Jawaid et al. demonstrated this, where 2 nm AuNPs were shown to decrease the intracellular levels of GSH, which led to an increase in intracellular ROS [57]. Similarly, Abudayyak et al. reported that Bi_2_O_3_ NPs also facilitated a decrease in the intracellular levels of GSH for various cell lines [58]. Regarding GSH, another reducing agent that has also been shown to be affected by NPs is thioredoxin reductase 1 (TrxR1). Similar to GSH, TrxR1 is important in maintaining redox equilibrium, regulating cellular redox processes, with its activity having been shown to be inhibited in the presence of Au clusters, leading to an increase in apoptosis [59]. Collectively, these studies suggest that NPs have the capacity to inhibit certain redox mechanisms and elevate ROS production, particularly in conjunction with radiotherapy. However, the full extent and implication of these interactions in which they contribute to the radiosensitization of cells remains to be elucidated.

### 3.3. Cell Cycle Effects

The cell cycle is a key determinant in the radiosensitivity of cells, with distinct phases demonstrating varying degrees of sensitivity. In general, cells in the late G2 and mitosis (G2/M) phase of the cycle exhibit enhanced sensitivity to radiation, whereas cells in the late S phase are markedly more radioresistant. Furthermore, the cell cycle is also integral to the DNA damage response following exposure to ionizing radiation, where the activation of cell cycle checkpoints is the second major effector pathway in the DNA damage response of cells [2]. These checkpoints either slow or halt the progression of cells through the cell cycle, maintaining genomic integrity by repairing radiation-induced damage or initiating apoptosis, thus preventing the replication of damaged DNA.

NPs have been shown to alter the cell cycle, with some studies demonstrating an enhancement in the population of the cells in the G2/M phase, causing a potential increase in the radiosensitivity of cells (Figure 5). Roa et al. observed that glucose-coated AuNPs increased the G2/M phase population 24 h after exposure to AuNPs [60]. This effect was linked to the activation of cyclin-dependent kinases, causing the acceleration of DU-145 cells through the G0/G1 phase towards G2/M, thereby augmenting their radiosensitivity. Similarly, Wang et al. observed that glucose-capped AuNPs arrested MDA-MB-231 cells in the G2/M phase, resulting in a sensitization enhancement ratio of 1.86 [61]. However, not all studies agree on the impact that NPs have on the cell cycle, with some research revealing negligible effects [62,63,64], which could be due to the effects of NPs varying for different cell types, as well as the different distinct physicochemical properties of NPs. Homila et al. studied the effects of AgNPs on a panel of lung cell lines, and their results found that AgNPs enhanced the accumulation of cells in the G2/M phase in both A549 and Calu-1 cell lines, while inducing S-phase accumulation in BEAS-2B cells and showing no effect on NCI-H358 cells [65]. Regarding physicochemical properties, the functionalization of NPs has also been shown to influence cell cycle distribution, where bovine serum albumin (BSA)-coated AuNPs induced G2/M arrest in RAW264.7 cells, contrasting with the G0/G1 arrest caused by non-functionalized AuNPs [66]. Together, these studies suggest that the alteration of the cell cycle induced by NPs could be dependent on both cell type and the properties of NPs, leading to the different results observed by various studies.

However, given the diversity in the physicochemical properties of NPs and the broad range of cell lines that have been studied, it is difficult to draw general conclusions on the effect that NPs have on the cell cycle and its contribution to the radiosensitization of cells; thus, further research is required to elicit definitive answers to understand how NPs and their properties can cause alterations. Nevertheless, existing studies indicate that NPs can modify the cell cycle distribution to enhance the sensitivity of cells to radiotherapy, where further research could elucidate ways to exploit this mechanism for therapeutic purposes.

## 4. Application of Nanoparticles as Radiosensitizers

### 4.1. High-Z Nanoparticles as Radiosensitizers

The use of high-Z nanoparticles as radiosensitizers has emerged as a novel tool in radiotherapy treatment. Initially, high-Z materials were proposed as radiosensitizers due to their potential to enhance dose deposition, a concept based on the increased X-ray absorption correlating with the atomic number of the absorbing material. This was first demonstrated using iodine (Z = 53) as a radiosensitizer, where Matsudaira et al. observed that iodine augmented the effects of X-rays in cell cultures over 40 years ago [67]. Following that study, it was shown that injecting iodine directly into tumors prior to RT suppressed tumor growth by 80% [68]. Together, these studies effectively demonstrated the feasibility of augmenting the effects of radiotherapy through the introduction of high-Z absorbers into target sites. Since then, advancements in nanotechnology have propelled the application of high-Z NPs as radiosensitizers. These NPs also aim to exploit the X-ray absorption phenomenon to augment the effects of radiotherapy, but they have also been shown to increase ROS production and alter the cell cycle dynamics, thereby enhancing the radiosensitivity of cells. Moreover, their physicochemical properties can be tailored for better biocompatibility, prolonged circulation time, and tumor targeting, making them suitable candidates as radiosensitizers.

Among other nanoparticles, AuNPs have been the most widely investigated in the field of high-Z NPs. A pioneering study conducted by Hainfeld et al. revealed their significant potential as radiosensitizers, where it was shown that injecting 1.9 nm AuNPs into mice bearing subcutaneous EMT-6 mammary carcinomas prior to 250 kVp X-ray irradiation significantly improved the one-year survival from 20% with radiotherapy alone to 86% with the combined treatment [69]. These promising results were also replicated in further studies using head and neck squamous carcinoma (SCCVII) and malignant glioma (Tu-2449) tumor models, providing compelling evidence about the potential applicability of AuNPs in radiotherapy treatment [70,71]. In these studies, mice were injected with AuNPs at a concentration ranging from ~2 to 4 g Au/kg body weight of mice. It was initially thought that such concentrations were required to achieve significant radiosensitization, which raised questions about the clinical translation of the strategy due to economic and safety concerns. Subsequent research, however, has indicated that substantial radiosensitizing effects can be realized at much lower AuNP concentrations, making the approach more viable for clinical use.

**Figure 6:**
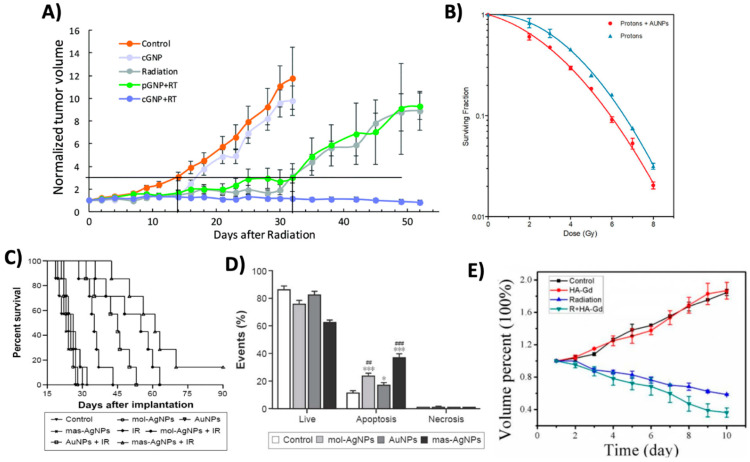
Radiosensitization due to high-Z nanoparticles. (**A**) Normalized tumor volumes of MDA-MB-231 xenograft tumor-bearing mice post treatment with cGNP alone, radiation (10 Gy single dose) alone, pGNP + radiation (RT), or cGNP + RT (**B**) Survival fraction curves of CHO-K1 cells treated with AuNPs and irradiated at various doses with a 200 MeV proton beam. (**C**) Kaplan–Meier survival curves for U251 tumor-bearing mice treated with AuNPs or AgNPs with or without ionizing radiation (IR). Mice were irradiated with a single dose of 8 Gy with a 6 MV X-ray beam. Mice were treated with AgNPs at the same molar concentration (mol-AgNPs) or mass concentration (mas-AgNPs) as AuNPs. (**D**) Apoptosis of U251 cells after treatment with AgNPs or AuNPs combined with a radiation dose of 6 Gy. * *p* < 0.05, *** *p* < 0.001 compared with the control group. ## *p* < 0.01, ### *p* < 0.001 compared with the corresponding AuNPs-treated group (**E**) Tumor volume growth curves after treatment with HA-Gd_2_O_3_ NPs and radiation (R + HA-Gd). Mice were administered 3 fractions of 3 Gy with a 6 MeV beam over a 7-day period for a total of 9 Gy. Reproduced with permission from open access Creative Common license [28,74,75,76].

A study by Liu et al. demonstrated the effectiveness of AuNPs at significantly reduced concentrations using bovine serum albumin (BSA)-functionalized AuNPs [72]. For their study, mice bearing H22 hepatoma tumors received injections of 4 mg Au/kg body weight, a thousand-fold reduction compared with the aforementioned studies. Their findings revealed that both 8 and 50 nm BSA-AuNPs suppressed tumor growth when used in conjunction with radiotherapy (5 Gy, 6 MV), achieving enhancement factors of 1.93 and 2.02, respectively. Similar results were also found by Bhattari et al., where they showed significant reductions in tumor growth in MDA-MB-231 xenograft models after a combined treatment of anti-CXCR4-functionalized GNPs (cGNP) and radiotherapy using a concentration of 2 mg Au/kg body weight [28]. This treatment led to a significant reduction in tumor growth, where tumor size reduced to baseline levels and regrowth did not occur for over 50 days post-treatment (Figure 6A). However, these radiosensitization effects were not realized with non-targeted PEGylated GNPs (pGNP), underscoring the importance of tumor-targeted NPs for radiosensitizing purposes.

Beyond conventional X-ray irradiation, the effectiveness of AuNPs can also extend to other radiation types. Electron radiotherapy, which is generally used for the treatment of superficial tumors, has been shown to benefit from AuNPs. Mehrnia et al. demonstrated this, where AuNPs under 4 MeV electron irradiation significantly decreased cell viability in both MCF-7 and MDA-MB-231 cells, with sensitizer enhancement ratios of 1.43 and 1.62, respectively [73]. These radiosensitizing effects were also observed in B16F10 melanoma tumor-bearing mice, where AuNPs significantly enhanced apoptotic cell death when irradiated with a 6 MeV electron beam, inducing the retardation of tumor growth [49]. Additionally, while not a common form of treatment, AuNPs have been shown to augment proton therapy. In a proof of principle study by Cunningham et al., AuNPs were found to increase cell death in CHO-K1 cells under 200 MeV proton irradiation by 27% and 44% at doses of 2 Gy and 6 Gy, respectively (Figure 6B) [74]. Collectively, these studies outline the broad application of AuNPs in radiotherapy, showcasing their potential to augment the effects of various types of radiation therapies.

Similar to gold, other high-Z nanoparticles, such as AgNPs, GdNPs, and BiNPs, have been explored as radiosensitizers. For instance, Liu et al. investigated the impact of AgNPs and radiotherapy in C6 glioma-bearing rats [77]. With the combined treatment, they observed anti-proliferative and pro-apoptotic effects, markedly improving survival times compared with the other treatment groups. Building on this research, they found similar results using a U251 glioblastoma mouse model [75]. Interestingly, their study displayed that AgNPs outperformed AuNPs in radiosensitization at equivalent concentrations (Figure 6C). It was reported that AgNPs significantly increased autophagy levels and pro-apoptotic activity (Figure 6D) when paired with radiation in comparison to AuNPs, which was attributed to the enhancement in the therapeutic results. Additionally, Gadolinium NPs (GdNPs) have been shown to be effective radiosensitizers, but they are also effective MRI contrast agents, enabling them to be a valuable tool for theranostics, which has led to their current clinical investigation. In a study by Wu et al., they found that hyaluronic acid-functionalized gadolinium oxide NPs (HA-Gd_2_O_3_ NPs) were effective in both roles [76]. The radiotherapeutic results displayed that HA-Gd_2_O_3_ NPs showed efficient suppression of tumor growth when combined with RT, with a 62% reduction in tumor volume 10 days post treatment (Figure 6E). Furthermore, studies have also demonstrated the potential radiosensitizing effects of BiNPs. A study by Deng et al. indicated that Folate-modified BiNPs enhanced the radiotherapeutic effects in 4T1 tumor-bearing mice, reducing the tumor growth and increasing the survival time compared with control groups without inducing any apparent toxicity, indicating the safety and efficacy of the treatment strategy [78].

### 4.2. Targeted Delivery of Radiosensitizing Chemotherapeutics via High-Z Nanoparticles

While radiotherapy predominantly aims to enhance local tumor control, chemotherapy is generally used to target metastasis, with the combination of these treatments becoming increasingly more common in the management of patients with locally advanced disease. In addition, various chemotherapeutic agents are known to augment the effects of radiotherapy, resulting in synergistic therapeutic benefits that can be exploited to improve patient outcomes. This concomitant use of radiotherapy and chemotherapy is a strategy that is applied in certain cancer treatment regimens, aiming to capitalize on the complementary mechanisms of both treatments to achieve better tumor control and curative rates, where studies have demonstrated increased loco-regional control and overall survival of patients [79,80,81,82]. However, issues that are innate to chemotherapeutics limit the overall efficacy of the treatment. Often, these drugs have poor solubility and a short circulation time, resulting in suboptimal concentrations reaching tumor sites. Furthermore, the lack of specificity causes systemic toxicity, inducing adverse effects that restrict the amount of dose that can be administered to patients.

In recent years, the advent of nano drug delivery systems (DDSs) has presented a solution to mitigate these challenges. Through encapsulation or the adsorption of drugs onto their surface, NPs can improve the pharmacokinetics, pharmacodynamics, and the targeted delivery of chemotherapeutics, ensuring that higher concentrations are delivered to tumors while simultaneously lowering normal tissue toxicity. The implementation of nano DDSs has already been proven to be effective, with nano DDSs such as Doxil (liposomal doxorubicin) and Abraxane (nab-paclitaxel) currently used in clinical settings [83]. Extending from this, the coupling of radiosensitizing chemotherapeutic agents with high-Z nanoparticles has been proposed to produce nanomedicines with dual-radiosensitizing effects, streamlining the therapeutic process.

Utilizing high-Z nanoparticles as DDSs for radiosensitizing agents presents a unique opportunity to introduce a bi-functional nanoplatform to improve cancer therapeutics. As previously discussed, high-Z nanoparticles inherently augment the effects of radiotherapy, and their combination with drug-based radiosensitizers can offer a dual sensitization effect that can be used to increase tumor responses to radiotherapy treatment. Their high surface area-to-volume ratio and adaptable surface chemistry facilitate the straightforward conjugation of moieties to their surface, including chemotherapeutics, and by leveraging their intrinsic therapeutic benefits, such as their tumor targeting and extended circulation time, drug delivery to tumors can be improved, leading to favorable therapeutic effects.

**Figure 7:**
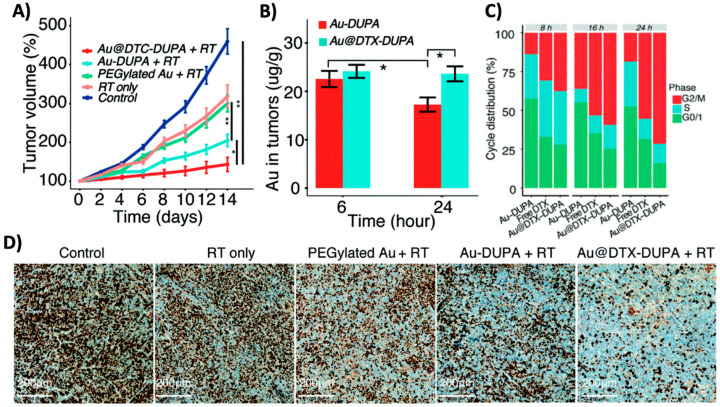
Dual radiosensitization through docetaxel-loaded gold nanoparticles. (**A**) Normalized tumor growth curves of 22RV1 xenograft tumor-bearing mice after irradiation of 6 Gy with 6 MeV electron beam. (**B**) Concentration of Au in 22RV1 xenograft tumor post-injection with Au-DUPA NPs or Au@DTX-DUPA NPs. (**C**) Cell cycle analysis of 22RV1 cells after treatment with Au-DUPA NPs, free DTX, or Au@DTX-DUPA NPs. (**D**) Ki67 staining assay of tumors 15 days post treatment to indicate proliferative capacity. * *p* < 0.05, ** *p* < 0.01. Reproduced with permission from open access Creative Common license [90].

One chemotherapy agent that has been delivered using high-Z NPs is docetaxel (DTX). DTX is a member of the taxane family that is used to effectively treat various forms of cancer, including head and neck, breast, and prostate. Its mechanism of action involves stabilizing microtubules within cells, thereby preventing the depolymerization required to progress through the cell cycle and undergo cell division, causing cells to be arrested in the G2/M phase. This phase is notably susceptible to radiation-induced damage, positioning DTX as an ideal radiosensitizer, with studies demonstrating synergistic effects between DTX and radiotherapy [84,85]. Additionally, DTX has been shown to increase NP accumulation in cells, both in vitro and in vivo, suggesting enhanced radiosensitization potential when used in combination with high-Z NPs [86,87,88,89]. A study by Guo et al. demonstrated the therapeutic prospect of synthesizing a AuNP-based DDS for DTX to enhance the effects of radiotherapy [90]. In their study, DUPA-modified AuNPs were loaded with DTX (Au@DTX-DUPA) to enhance the radiosensitization of tumors. Following treatment with Au@DTX-DUPA and radiotherapy, 22RV1 tumor-bearing mice demonstrated significant therapeutic benefits due to the sensitization effects of both DTX and AuNPs, with a significant reduction in tumor growth being observed for the combined treatment (Figure 7A). This effect could be attributed to the enhanced sensitization from the combination of both AuNPs and DTX. As shown in Figure 7B, there was an enhancement in AuNP accumulation in tumor tissue 24 h post-injection for mice treated with Au@DTX-DUPA, the timepoint at which mice were irradiated. Furthermore, Au@DTX-DUPA successfully induced cell arrest in the G2/M phase of the cell cycle, indicating increased levels of sensitization (Figure 7C). This resulted in a significant decrease in Ki67 expression for the combined treatment of Au@DTX-DUPA and radiotherapy, signaling a significant inhibition of the proliferative capacity of tumors (Figure 7D) and highlighting the promising capabilities of high-Z NPs as a dual-sensitizing DDS.

Similar to DTX, cisplatin is another chemotherapeutic agent known to enhance cell sensitivity to radiotherapy. The platinum-based chemotherapeutic agent functions by binding to DNA, inducing both intrastrand and interstrand crosslinks. Like other chemotherapeutics, the efficacy of cisplatin is often compromised by systemic toxicity that stems from its lack of specificity. Therefore, implementing a DDS has the potential to mitigate this issue. A recent study by Chen et al. demonstrated this using AuNPs as a DDS for cisplatin [91]. It was noted that cisplatin in its free form caused acute systemic toxicity in mice, characterized by a significant decrease in body weight a week after injection, where this was not realized for mice treated with Au-cisplatin NPs. Furthermore, Au-cisplatin NPs were shown to also diminish nephrotoxicity and hepatotoxicity associated with cisplatin in its free form. When combined with ablative radiotherapy, Au-cisplatin NPs demonstrated enhanced radiosensitization effects, decreasing tumor growth and improving the overall survival of mice. It was also shown to elicit immunogenic cell death, which enhanced the recruitment of tumor-infiltrating immune cells, such as natural killer T cells and CD8+ T cells. Notably, this resulted in distant non-irradiated tumors experiencing growth suppression, showing the potential of Au-cisplatin NPs to prime the immune system to reduce the metastatic potential and induce abscopal effects.

### 4.3. Challenges and Considerations in the Application of High-Z NPs as Radiosensitizers

Despite the compelling evidence that promotes the application of high-Z NPs in radiotherapy treatment, there are still challenges that are apparent that can limit their clinical translation. Various factors and parameters, such as NP material, size, surface modifications, and concentrations need to be considered when designing NPs to obtain optimal results. However, when taking these factors into consideration, it is not abundantly clear as to which parameters will provide the best therapeutic results.

Many studies have shown a correlation between the size of NPs and their cellular uptake. An early study conducted by Chithrani et al. demonstrated a difference in the cellular uptake of GNPs with diameters ranging from 14–100 nm, with the results concluding that 50 nm GNPs had the greatest uptake [92]. Similar studies have also shown that ~50 nm diameter GNPs had a significant increase in uptake in human prostate carcinoma PC-3 cells in comparison with their smaller and larger counterparts, suggesting that 50 nm NPs are ideal to obtain optimal uptake, and thus radiosensitization [93]. Interstingly, when moving into more complex models, such as multilayer cell structures and 3D tumor spheroids, smaller NPs appear to be more advantageous. One study compared the uptake of 20 nm and 50 nm GNPs in tissue-like multilayer cellular structures that were grown to model post-vascular tumor environments [94]. Although uptake was greater for 50 nm GNPs at the monolayer level, 20 nm GNPs penetrated multilayer cellular structures more efficiently, suggesting a reversal in size dependency for multilayer structures. This has also been supported by uptake data from three-dimensional spheroids. GNPs of 15 nm increased the depth penetration by 33% and 17% for CAL-27 and HeLa spheroids, respectively, compared with 50 nm GNPs [95], which can be attributed to the extracellular matrix (ECM) and its composition. As NPs diffuse through the ECM, they interact with the network structure via hydrodynamic interactions, physical interactions, as well as electrostatic interactions that can constitute a barrier to some NPs. One of these barriers is the inter-matrix spacing in the ECM, an important component when considering the optimal size of NPs. Collagen fibrils that reside in the ECM present a size limit for NPs where particles cannot penetrate the matrix barrier if the diameters surpass the size of the network space. Despite this, smaller NPs (<10 nm) can also present some disadvantages. These smaller NPs can enter the nucleus of a cell, which may result in toxicity [96,97]. In addition, NPs with a size of <10 nm can also experience rapid clearance from the RES, resulting in inadequate delivery of NPs to target sites.

To achieve optimal therapeutic results, it is paramount to ensure that adequate concentrations of NPs in tumors are met to achieve radiosensitization, as more NPs present in the tumor will lead to a greater sensitizing effect. However, simply increasing the concentrations administered can lead to an increased risk of toxicity. Biodistribution studies have demonstrated the accumulation of NPs in organs such as the liver, spleen, and kidneys, raising concerns about their use [98]. As such, the safety and biocompatibility of NPs should be of utmost importance to avoid acute and late toxicities that could hinder their therapeutic efficacy. As a result, many preclinical studies focus on exploring the surface modification of NPs to improve their biocompatibility, circulation time, and targeting. A wide variety of functionalization strategies have been explored in preclinical studies, with most suggesting beneficial therapeutic results. However, this poses a challenge in itself to pinpoint what array of NPs are the most beneficial, or rather most clinically feasible. Perhaps one aspect that should be prioritized when deciding this is their ability to be produced on a large scale that is required for clinical applications. Although studies demonstrate tremendous success in synthesizing and functionalizing a wide variety of NPs, the commercialization required when transitioning from bench to bedside necessitates large scaled-up synthesis that can present obstacles, such as reproducibility and quality control. This begs the question of whether the design of certain NPs used as radiosensitizers is for academic purposes or for clinical intentions. The synthesis of NPs at the lab scale allows for the precise synthesis conditions required to produce highly uniform and reproducible NPs. On the other hand, scaled-up synthesis often can introduce variability, resulting in variations in the properties of the NPs. Therefore, it is important to consider the feasibility of using cost-effective, well-controlled, and good manufacturing practices to produce large reproducible batches of NPs, and this should be considered early on in preclinical studies to ease their clinical translation.

### 4.4. Clinical Translation of High-Z Nanoparticles as Radiosensitizers

Although preclinical studies have suggested that the application of high-Z nanoparticles in radiotherapy can provide significant benefits, their clinical translation remains limited in comparison with more commonly studied organic NPs, such as liposomes and polymers. High-Z NPs encounter distinct challenges in terms of their clinical adaptation, including concerns regarding their biocompatibility, toxicity, biodistribution, and the complexities associated with scaled-up synthesis. Despite this, two radiosensitizing high-Z NPs, NBTXR3 and AGuIX, have transitioned into clinical trials, indicating a potential shift in the clinical application of high-Z NPs as radiosensitizers.

NBTXR3, developed by Nanobiotix, is a hafnium oxide (HfO_2_) NP currently under clinical investigation. Similar to other high-Z nanoparticles, its radiosensitizing abilities arise from hafnium’s high atomic number (Z = 72), increasing dose deposition via physical mechanisms. When present in tumors, hafnium increases the interactions with ionizing radiation, yielding a local enhancement in the generation of dose-depositing electrons and DNA-damaging free radicals, thereby amplifying DNA damage and the cytotoxicity beyond that of conventional radiotherapy. Currently, clinical studies are investigating NBTXR3’s radiosensitizing effects across various types of cancers, including soft tissue sarcoma, head and neck, prostate, lung, and brain metastases (Table 1). Interestingly, in contrast to many preclinical studies that examined the efficacy of high-Z NPs as radiosensitizers, these clinical trials are analyzing the effects of intratumoral injections of NBTXR3 combined with radiotherapy, a strategy that might ease the clinical translation of radiosensitizing NPs. Intratumoral injections address concerns such as systemic toxicity and biodistribution issues that are associated with intravenous injections, where NPs often accumulate in organs like the kidneys, liver, and spleen, despite their functionalization with targeting moieties [87,88,89]. This raises concerns about the toxicity of the treatment, where lower concentrations would need to be administered at the cost of reducing dose enhancement. Intratumoral injections can help to mitigate this trade-off, enabling higher concentrations in tumors without increasing the risk of toxicity. A recent phase II/III clinical trial (NCT02379845) has shown encouraging results, with intratumoral injections of NBTXR3 demonstrating increased anticancer effects and tolerability in patients with locally advanced soft-tissue sarcoma [99]. That study found an increase in complete pathological response for patients who received NBTXR3 in conjunction with preoperative radiotherapy compared with preoperative radiotherapy alone, suggesting promising therapeutic benefits of incorporating high-Z NPs to complement the effects of radiotherapy. Additionally, preclinical studies have suggested that NBTXR3 can enhance the efficacy of immunotherapies by bolstering the antitumor immune response, prompting clinical investigations of the combined treatment modalities (NCT05039632, NCT04862455, NCT03589339, and NCT04892173) [100,101,102].

In addition to NBTXR3, AGuIX, developed by NH TherAguix, is another nanoparticle currently under clinical investigation. These sub 5 nm nanoparticles are composed of gadolinium (Z = 64)-chelated polysiloxane, where the strategic choice of gadolinium enables them to be effective radiosensitizers and MRI contrast agents. When administered intravenously to patients, AGuIX can preferentially accumulate in tumors due to the EPR effect, allowing for enhanced concentrations in tumor tissue compared with the surrounding healthy tissue. Furthermore, their small size promotes effective renal clearance, avoiding potential toxicity that could be induced by long-term retention [103]. Upon irradiation, the Gd chelates enhance the local dose deposition through increased interactions with ionizing radiation, where preclinical studies have demonstrated favorable radiotherapeutic effects [104,105,106]. Additionally, the enhanced MRI contrast provided by the accumulation of AGuIX NPs in tumor sites facilitates better imaging, improving the quality of treatment planning and real-time patient monitoring that can be used to improve adaptive radiotherapeutic techniques. A current clinical trial is aiming to exploit these features, coupling AGuIX with stereotactic magnetic resonance-guided adaptive radiation therapy (SMART) for the treatment of lung tumors and pancreatic cancer (NCT04789486). This cutting-edge delivery technique is designed to provide high-resolution, real-time MR imaging to ensure the precise delivery of stereotactic radiation therapy, thus leveraging AGuIX’s image enhancement and dose deposition capabilities to potentially further improve patient outcomes. Along with this study, there are other clinical trials that are investigating the effects of AGuIX and radiotherapy for various types of cancer as shown in Table 1. Despite these studies being in their early stages, results from a recent phase I clinical study (NCT02820454) indicated AGuIX’s safety and tolerability, along with potential clinical benefits when combined with radiotherapy [107].

## 5. Conclusions

The field of nanotechnology has promoted a transformative shift in cancer therapeutics, offering innovative approaches to enhance treatment efficacy and patient outcomes. Among these advances, the application of nanoparticles as radiosensitizers presents a promising frontier to improve radiotherapy treatment. High-Z nanoparticles have emerged as a novel solution to overcome challenges inherent to radiotherapy, such as normal tissue toxicity and radiation resistance of tumors. Their unique physico-chemical properties can be tailored to preferentially target tumors, maximizing the therapeutic impact on tumors without inducing additional damage to surrounding healthy tissue. Preclinical studies have already demonstrated the significant therapeutic benefits that high-Z nanoparticles can provide, including their potential application as drug delivery systems to further augment the effects of radiotherapy. Yet, the clinical application and efficacy of high-Z NPs remain to be fully investigated, with few clinical trials exploring the effects of high-Z NPs as radiosensitizers. This could be due to several factors, such as the lack of understanding behind the mechanisms of sensitization, safety concerns, and the challenges in the up-scaled synthesis of NPs required for clinical applications. However, as further research is conducted on NPs, it will elicit a better understanding on their mechanisms of sensitization, their interactions with biological systems, and their potential toxicities, enabling the production of optimized nanoparticle systems to take full advantage of their abilities to improve radiotherapy. Furthermore, encouraging results in the current clinical trials will provide a rationale for high-Z NPs to improve radiotherapy, and may result in a shift towards more high-Z NPs being clinically investigated, laying the foundation required to transition their use into radiotherapy treatment regimens to improve the prognosis for cancer patients.

## Figures and Tables

**Table 1:** Current clinical studies investigating the effects of high-Z NPs for radiotherapy.

Nanoparticle	Condition	Phase	Status	Identifier
NBTXR3	Adult soft-tissue sarcoma	Phase I	Completed	NCT01433068
	Pancreatic ductal adenocarcinoma	Phase I	Recruiting	NCT04484909
	Lung non-small-cell carcinoma	Phase I	Recruiting	NCT04505267
	Metastatic malignant solid neoplasm	Phase I/II	Recruiting	NCT05039632
	Esophageal adenocarcinoma	Phase I	Recruiting	NCT04615013
	Head and neck squamous cell carcinoma	Phase II	Recruiting	NCT04862455
	Advanced cancers	Phase I	Recruiting	NCT03589339
	Head and neck squamous	Phase I	Active	NCT01946867
	Adult soft-tissue sarcoma	Phase II/III	Completed	NCT02379845
	Head and neck squamous cell carcinoma	Phase III	Recruiting	NCT04892173
AGuIX	Glioblastoma	Phase I/II	Recruiting	NCT04881032
	Brain metastases	Phase I	Completed	NCT02820454
	Brain metastases	Phase II	Recruiting	NCT03818386
	Gynecological cancers	Phase I	Recruiting	NCT03308604
	Brain metastases	Phase II	Recruiting	NCT04899908
	Lung tumors and pancreatic cancer	Phase I/II	Recruiting	NCT04789486
	Recurrent cancer	Phase I	Not yet recruiting	NCT04784221

## Data Availability

Data sharing is not applicable.
